# Oculomotor and Vestibular Deficits in Friedreich Ataxia - Systematic Review and Meta-Analysis of Quantitative Measurements

**DOI:** 10.1007/s12311-024-01716-8

**Published:** 2024-07-27

**Authors:** E. Sohns, D. J. Szmulewicz, A. A. Tarnutzer

**Affiliations:** 1 Faculty of Medicine, University of Zurich, Zurich, Switzerland; 2 Balance Disorders & Ataxia Service, Royal Victorian Eye and Ear Hospital, Melbourne, VIC Australia; 3 The Bionics Institute, Melbourne, VIC Australia; 4 University of Melbourne AU, Melbourne, VIC Australia; 5 Neurology, Cantonal Hospital of Baden, Im Ergel 1, Baden, 5404 Switzerland

**Keywords:** Hereditary ataxia, Eye movements, Quantitative analysis, Vestibular, Oculography

## Abstract

Disease-specific oculomotor assessments play a crucial role in the early diagnosis of hereditary cerebellar ataxias. Whereas several studies have reported on quantitative oculomotor and vestibular measurements in Friedreich’s Ataxia (FRDA), the value of specific oculomotor paradigms remains unclear. We aimed to address this knowledge gap through a systematic literature review and providing disease-specific recommendations for a tailored set of eye-movement recordings in FRDA. MEDLINE and Embase were searched for studies reporting on quantitative oculomotor and/or vestibular measurements in FRDA-patients. Data on oculomotor and vestibular parameters were extracted and correlations with a range of clinical parameters were sought. Included studies (*n* = 17) reported on 185 patients. Abnormalities observed included the presence of saccadic intrusions (143/161) such as square-wave jerks (SWJ, 90/109) and ocular flutter (21/43), impaired eccentric gaze-holding (40/104), abnormal pursuit (81/93) and angular vestibulo-ocular reflex (aVOR) deficits (39/48). For visually-guided saccades (VGS), we frequently observed increases in saccade latency (27/38) and dysmetric saccades (71/93), whereas saccade velocity was more often preserved (37/43). Augmented anti-saccade (AS) latency, downbeat nystagmus and frequent macro-SWJ correlated with disease duration. Increased AS-latency and VGS-latency, frequent macro-SWJ, reduced aVOR-gain and augmented aVOR peak-latency correlated with disease severity. A broad range of oculomotor and vestibular deficits are documented in the literature. Impairments in pursuit, saccades and aVOR-responses are most commonly reported, and as such, should be prioritized as disease markers. Quantitative oculomotor testing in FRDA may facilitate early diagnosis and prove valuable in monitoring disease progression and treatment response.

**Supplementary Information:**

The online version contains supplementary material available at 10.1007/s12311-024-01716-8.

## Introduction

Friedreich’s ataxia (FRDA) is an autosomal recessive neurodegenerative disease. It represents one of the most frequent hereditary ataxias, as approximately 1 in every 20,000 to 1 in every 125,000 individuals within different Western European populations are estimated to be affected by the disease, with an equal distribution between men and women [1]. The mutation involved is generally a GAA-triplet expansion in the FXN gene, which codes for frataxin, a key protein in mitochondrial metabolism [2]. The reduction in frataxin results in a disruption of the iron-sulfur cluster synthesis, leading to ATP production impairment. In the context of FRDA, the production of reactive oxygen species could play a central role in the mechanism of the disease [2]. FRDA affects the central and peripheral nervous system, causing characteristic progressive clinical conditions such as gait ataxia, sensory impairment, muscle weakness, dysarthria, dysphagia, visual and hearing deficits, eye movements abnormalities and cognitive impairment [3]. In addition to neurological manifestations, FRDA may also involve other organ systems, resulting in cardiomyopathy, kyphoscoliosis or diabetes [3]. Onset of FRDA occurs between the ages of 5 and 20 years of age, but it can present outside this range with some patients presenting as late as their 60s [4]. Noteworthy, late-onset variants with appearance of first symptoms after the age of 25 years occur in 17% of cases [5]. Life expectancy is reduced to an average of 45 years, although people have survived out the eighth decade [6]. In advanced stages, the disease can lead to atrophy of the spinal cord and the cerebellum [7]. In early onset FRDA (< 15 years), wheelchair dependence occurs at a median 11.5 years after symptom onset [8]. There is currently no curative therapy for FRDA, however, recently omaveloxolone - a potent activator of nuclear factor erythroid 2-related factor 2 (NRF2) – has been shown to significantly improve neurological function [9] and thus has been approved as the first treatment for FRDA by the US Federal drug Administration (FDA) and the European Medicines Agency (EMA). In addition to surveillance for diabetes mellitus, scoliosis, cardiac arrhythmias, and progressive cardiomyopathy, the development of further targeted treatments is needed to both significantly lengthen a patient’s life expectancy and enhance their quality of life [10].

The diagnosis of FRDA is suspected in those with a history of slowly progressive cerebellar ataxia and in light of the autosomal recessive inheritance pattern, regardless of the existence of a positive family history [6]. In such an individual, the index of diagnostic suspicion may be heightened by oculomotor findings which display a combination of abnormalities including square-wave jerks (SWJ), abnormal pursuit eye movements (PEM), dysmetric saccades to target and others [11]. The diagnosis of FRDA may at times be delayed due of factors such as limited clinical awareness and non-specific initial symptoms [3]. An oculomotor assessment represents a key element in formulating the differential diagnosis of a patient presenting with cerebellar ataxia. The definitive diagnosis of the disease is established via molecular genetic testing [6].

In the past, various oculomotor and vestibular signs in patients with FRDA have been described at the bedside and also quantitatively by use of electronystagmography (ENG) and subsequently, video-oculography (VOG). This includes abnormalities in PEM, saccades to target, gaze holding, and the angular vestibulo-ocular reflex (aVOR) [11]. Most studies reported single cases or small case series, using different experimental paradigms and methodologies. A systematic review or meta-analysis of quantitative oculomotor and vestibular abnormalities in FRDA has not to our knowledge hitherto been performed. Although genetic diagnosis is based on established standards [6], no standards exist for the objective characterization of disease stage or severity. A disease-specific eye movement recording paradigm would potentially offer a robust, objective means of monitoring disease progression, particularly in early disease stages as well as a sensitive means of early disease detection [12]. We therefore aimed to address this knowledge gap in the literature by undertaking a systematic review in order to summarize quantitative oculomotor and vestibular abnormalities observed in FRDA. This will offer more detailed knowledge about oculomotor patterns observed and frequencies of eye movement abnormalities reported in FRDA. Employing this information we then aimed to formulate disease-specific recommendations for a tailored set of eye movement recording paradigms for FRDA. Additionally, we sought to identify possible correlations between oculomotor findings and variables such as disease duration, repeat length or clinical scores.

## Methods

### Data Sources and Searches

We searched MEDLINE and Embase for English-language articles, relying on the following strategy and looking for specific components in all articles: (1) defining FRDA, (2) oculomotor or vestibular features, and (3) quantitative assessments. We then selected a series of textual terms to enter in the search system that would refer to the selected criteria. We did not specifically conduct searches for hereditary ataxia syndromes, as doing so led to the exclusion of many relevant studies, primarily because they did not explicitly reference the genetic aspect. A manual search of the references of eligible articles was also performed and we contacted corresponding authors, where necessary. We did not seek to identify research abstracts from meeting proceedings or unpublished studies. Since the submitted work is a systematic review, ethical approval was not necessary.

### Study Selection and Quality Assessment

We used predetermined inclusion criteria and a controlled methodology to select the relevant studies. This was conducted by two independent raters (ES and AAT). Differences were resolved by discussion and consensus. We calculated inter-rater agreement on full-text inclusion using Cohen’s kappa [13]. Only English-language articles with original data on human subjects with FRDA, reporting quantitative oculomotor and/or vestibular measurements were included. The search strategy was designed by a clinical investigator with relevant domain expertise in neurology (AAT).

**Fig. 1:**
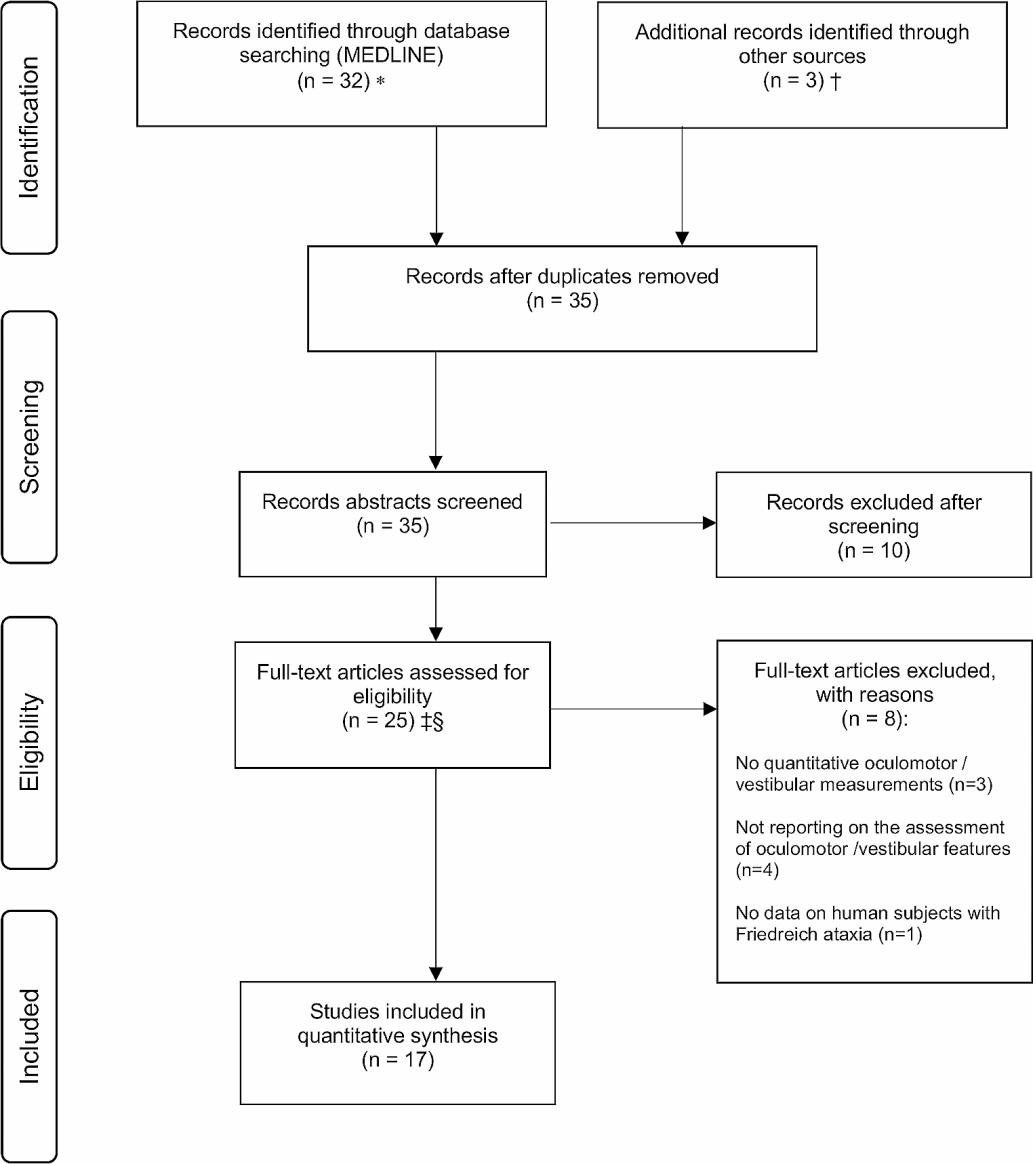
PRISMA flow chart. * MEDLINE was accessed via PubMed. † Additional literature research based on a recent systematic review on oculomotor assessments in hereditary ataxia from the same group [11] and on a recent systematic review on neuro-ophthalmological findings in FRDA [14]. ‡ Abstracts coded as ”yes” or ”maybe” by at least one reviewer were included in the full-text review. § In cases of different evaluation, a final decision was made through discussion and consensus between the two reviewers

Our search identified 32 unique citations and 3 additional records were included after a literature search based on a recent systematic review of the oculomotor assessment in hereditary ataxias from our group [11] as well as a recent systematic review on neuro-ophthalmological findings in FRDA [14]. Of 35 papers screened, 10 (29%) were excluded at the abstract level (see PRISMA flow chart in Fig. 1). A record was excluded if two raters recommended exclusion (for detailed list of predefined reasons for exclusion see Appendix 1). We further examined 25 manuscripts at the full-text level. While 17 were considered eligible, 8 were excluded for the following reasons: 3 did not report quantitative oculomotor or vestibular measurements, 4 did not report on the assessment of oculomotor or vestibular features, and 1 did not contain data on human subjects with FRDA.

An assessment of the study quality of the examined publications was conducted using eight predetermined criteria covering factors related to: (i) study cohort, (ii) data acquisition, and (iii) data analysis (see Appendix 2) that were previously proposed by our group [11]. Based on this process, a comprehensive determination of study quality (categorized as high, moderate, or low) was established.

### Data Extraction, Synthesis, and Analysis

For each eligible study, information about oculomotor and/or vestibular parameters reported, including PEM, saccadic eye movements (SEM), gaze-holding, optokinetic nystagmus (OKN) and the aVOR, were actively searched for and extracted. Subsequently, we determined the frequency of presentation and the degree of abnormality for each oculomotor/vestibular parameter identified. For the frequency fraction calculation, only studies with frequency values or single patients’ values were considered, whereas for the degree of abnormality determination, studies with single patients’ values or mean values were contemplated. The frequency of presentation was then graded by symbols (from “-“ to “+++”), each corresponding to a specific percentage range of presentation (see caption of Tables 1 and 2 for details). As regards the level of abnormality, the difference between the mean value of the patient group and that of the control group was calculated. The variation in percentage was graded from “↑↑↑” to “↓↓↓”, similarly to the frequency fractions (see captions of Tables 1 and 2 for details). Where possible, a meta-analysis was performed. For most publications considered, this was not possible due to differing methodologies in oculomotor measurement paradigms. We also considered potential correlations between oculomotor/vestibular parameters and other variables, such as disease-duration, age of symptom-onset and clinical scores, (e.g. Friedrich’s Ataxia Rating Scale (FARS), Sloan Low Contrast Letter Chart (SLCLC) and Scale for the Assessment and Rating of Ataxia (SARA)). While the SLCLC specifically investigates visual function [15], the FARS assesses bulbar function, upper limb, lower limb, activities of daily living, upright stability and gait functions [16]. Similarly, the SARA examines gait, stance, sitting, speech disturbance, upper and lower limb [17]. SWJ were categorized as micro-SWJ (amplitude < 0.5°), SWJ (amplitude between 0.5° and 3°) or macro-SWJ (amplitude between 3° and 30°) [15].

**Table 1:** Overview of oculomotor findings – level of abnormality and frequency of presentation

	Level of abnormality	*n*	Frequency of presentation*	*n*
**Pursuit eye movements**			+++ [15, 18–25]	93
Gain	↓ † [18–20, 26]	58	+++ [15, 18, 19]	40
Catch-up saccades	-		+++ [15, 21]	31
**Saccadic eye movements**				
VGS - latency	↑↑ [15, 18, 27, 28]	49	+++ [18, 26]	29
VGS – velocity	(↓) § [15, 18–, 20, 22, 23, 26–28]	110	=/+ [18, 19, 22, 23, 27]	43
VGS - accuracy	(↓↓) [15, 19, 27, 28]	55	+++ [18–23, 25, 26]	93
Hypermetry	-		++ [18, 19, 26]	38
Hypometry	-		+ [18, 19, 26]	38
AS - latency	↑↑↑ [28]	13	-	
AS - accuracy	↓↓ ‡ [28]	13	-	
MSG - latency	↑↑↑ [28]	13	-	
MGS - accuracy	↓ ‡ [28]	13	-	
**Saccadic intrusions**	-		+++ [15, 18–27, 29]	161
Square-wave jerks	-		+++ [18, 19, 21, 22, 24, 26, 27, 29]	111
Ocular flutter	-		++ [19, 22, 26]	43
**Eccentric gaze holding**				
Gaze-evoked nystagmus	-		+ [15, 18–21, 23, 24, 26]	104
Rebound nystagmus	-		=/+ [18, 19, 21–23, 26]	69
**Spontaneous nystagmus**	-		=/+ [15, 18, 19, 23, 26]	70
**Positional nystagmus**	-		∅ [23]	5
**OKN impairment**	-		+ [18, 19, 21–24, 26]	68
Gain	↓ [18–20, 26]	58	+ [18, 19, 21–24, 26]	35
Slow-phase velocity	↓ [20]	16	+ [21–23]	24

Symbol legend:

Level of abnormality:

↑/ ↓ = reduced/increased by 5–25% (mildly)

↑↑/↓↓ = reduced/increased by 25–50% (moderately)

↑↑↑/↓↓↓ = reduced/increased by > 50% (strongly)

Frequency of presentation:

∅ = not present

=/+ = fraction 5–20% (rarely)

+ = fraction 20–40% (sometimes)

++ = fraction 40–70% (in a significant fraction)

+++ = fraction > 70% (frequently)

() = not calculated

n = number of patients undergone testing

* Frequency rates for a parameter refer only to studies presenting data from individual patients, not to the totality of studies in which it was measured

† 0.2 Hz sinusoidal movements

§ Difficult to determinate because of different measurement methods (10°, 20°, or 30° saccades)

‡ Determined through absolute position error

*Abbreviations* AS = anti-saccades; MGS = memory-guided saccades; OKN = Optokinetic nystagmus; VGS = visually-guided saccades

**Table 2:** Overview of vestibulo-ocular reflex findings – level of abnormality and frequency of presentation

	Level of abnormality	*n*	Frequency of presentation	*n*
**Low-frequency aVOR**	**-**		+++ [18, 19, 26, 30]	48
Gain	↓↓ [ 18–21, 26, 30]	59	+ [18, 19, 26, 30]	13
Time constant	↓↓ [18, 19]	19	++ [19]	12
Phase lead	↑↑↑ [26, 30]	24	+++ [26, 30]	24
**aVOR suppression**			++ [18, 19, 21, 22, 26]	61
Gain	↑↑↑ [26, 20]	44	+ [18, 26]	28
**High-frequency aVOR (vHIT)**				
Latency	↑↑↑ [15, 31]	24	-	
Gain	↓↓ [15, 31]	24	-	
**Caloric irrigation**	**-**		++ [19, 21–23]	37
Slow phase velocity	↓↓ [19]	12	+++ [19, 21]	27

Symbol legend:

Level of abnormality:

↑/ ↓ = reduced/increased by 5–25% (mildly)

↑↑/↓↓ = reduced/increased by 25–50% (moderately)

↑↑↑/↓↓↓ = reduced/increased by > 50% (strongly)

Frequency of presentation:

∅ = not present

=/+ = fraction 5–20% (rarely)

+ = fraction 20–40% (sometimes)

++ = fraction 40–70% (in a significant fraction)

+++ = fraction > 70% (frequently)

() = not calculated

n = number of patients undergone testing

*Abbreviations* aVOR = angular vestibulo-ocular reflex; vHIT = video head-impulse testing

## Results

### Overview of Studies

For the systematic review and meta-analysis, a total of 17 studies (all of them prospective and with single study locations in 15/17) were included. Overall study quality was high in 4 studies, moderate in 4 studies and low in 9 studies. Data on patients with FRDA were extracted. The diagnosis was either genetically confirmed (9/17 studies, 97 patients) or based on clinical features and/or a positive family history (8/17 studies, 88 patients). The mean GAA repeat length was 1206 ± 483 b for the small allele [15, 27–29], and 941 ± 147 b [15, 27] for the large allele. Electro-oculography (EOG) using electromyography (EMG) was most frequently used for recording eye movements; other devices used were scleral search coils (SSC) and video-oculography (VOG). Further information on the studies are listed in Table 3.

**Table 3:** Study designs and patient population

	Studies (*n*)	Patients (*n*)
**Study location**		
Monocentric	15 [18–24, 26–33]	164
Multicentric	2 [15, 25]	21
**Study type**		
Case series	7 [15, 21–25, 29]	86
Case control studies	10 [18–20, 26–28, 30–33]	99
**Patient population**		
Genetically confirmed diagnosis	9 [15, 18, 27–29, 30–33]*	97
Diagnosis based on clinic or family history	8 [19–26]	88
**Sex**		
Female patients included	9 [19–22, 24–27, 31]	50
Male patients included	8 [19–22, 24, 26, 27, 31]	54
Gender not reported	8 [15, 18, 23, 28, 29, 30, 32, 33]	81
**EM data collection**		
Electro-oculography (EOG)	10 [18–26, 29]	132
Scleral search coils	4 [28, 30, 32, 33]	15
Video-oculography (VOG)	2 [27, 31]	18
VOG and/or SSC	1 [15]	20

*Abbreviations* EM = Eye movement; SSC = scleral search coils; VOG = video-oculography

*In [18] no genetic testing was performed in 3/7 patients, but no subgroup analysis was possible

**Table 4:** Recorded oculomotor and vestibular parameters

	Studies (*n*)			Patients (*n*)		
	Horizontal EM only	Vertical and horizontal EM	Total	Horizontal EM only	Vertical and horizontal EM	Total
**Gaze holding**						
SN in primary gaze position	2 [18, 23]	3 [15, 19, 26]	5	7	60	67
Gaze-evoked nystagmus	4 [18, 19, 23, 24]	4 [15, 20, 21, 26]	8	27	77	104
Rebound nystagmus	3 [18, 19, 23]	2 [20, 26]	5	25	35	60
**OKN**	6 [18–20, 22, 23, 26]	2 [21, 24]	8	81	13	94
**Positional nystagmus**	1 [23]	-	1	5	-	5
**Pursuit eye movements**	7 [15, 18–20, 23, 25, 26]	3 [21, 22, 24]	10	92	23	115
**Saccadic eye movements**						
Visually-guided saccades	11 [15, 18–21, 23, 25, 26, 28, 32, 33]	2 [22, 27]	13	116	19	135
Memory-guided saccades	1 [28]	-	1	-	-	13
Anti-saccades	1 [28]	-	1	-	-	13
**Saccadic intrusions**	5 [18, 23–25, 29]	7 [15, 19–22, 26, 27]	12	52	109	161
**VOR**						
Caloric irrigation	-	-	4	-	-	39
Decay TC	2 [18, 19]	-	2	-	-	20
Rotational	6 [18–20, 23, 26, 30]	-	6	-	-	73
Translational	1 [30]	-	1	-	-	2
Vision-enhanced	1 [20]	-	1	-	-	24
VOR high-frequency	1 [32]	1 [15]	2	9	20	29
VOR-suppression	5 [18–20, 22, 26]	1 [21]	6	76	11	87
**Total EM recordings**	**10** [18, 23–25, 28–33]	**7** [15, 19–22, 26, 27]	**17**	**76**	**109**	**185**

*Abbreviations* EM = eye movement; OKN = Optokinetic nystagmus; PEM = pursuit eye movements; SN = Spontaneous nystagmus; TC = Time constant; VOR = Vestibulo-ocular reflex

### Overview of Recorded Oculomotor and Vestibular Parameters

Table 4 gives an overview of the number of studies reporting on oculomotor and vestibular parameters studied in FRDA. The most frequently reported oculomotor parameters in the studies included in our systematic review were SEM and saccadic intrusions (SI), which were generally measured in both the horizontal and the vertical planes (7 out of 12 studies). In contrast, the majority of visually-guided saccades (VGS) recordings were only captured in the horizontal plane.

### Oculomotor Findings

#### Pursuit Eye Movements (PEM)

PEM were altered in almost all patients across the 9 studies that reported a frequency of presentation of this parameter (87%, 81/93). Abnormalities reported included SI during pursuit (95%, 69/73 patients from 7 studies) and decreased pursuit gain (80%, 32/40 patients from 3 studies) [15, 18, 19], with two more studies reporting significantly reduced pursuit gain [20, 26] without specifying the frequency of presentation of gain reduction. Catch-up saccades were also observed (94%, 29/31), with only two studies reporting on the abnormality [15, 21]). When following a 0.2 Hz sinusoidal pursuit stimulus, gain was only moderately reduced (22.8% decrease on average) compared to the controls [18–20, 26]. The mean gain-value between two studies employing identical recording paradigms (sinusoidal movements at 0.2 Hz and ± 20° degrees of amplitude) was 0.79 (SD = 0) [18, 19]. Gain values in FRDA patients showed a tendency for more pronounced impairment at higher frequencies of sinusoidal pursuit stimuli compared to lower frequencies, specifically 0.1, 0.2 and 0.4 Hz [15, 26].

#### Saccadic Eye Movements

In two studies saccades were measured in both horizontal and vertical plane, but no differential effect was mentioned [22, 27].

##### Saccade Latency in Visually-Guided Saccades

Saccade latency was significantly prolonged in 76% (22/29) of FRDA patients studied (2 studies reporting on frequency fractions) [18, 26]. In three more studies significantly augmented latencies were reported [15, 27, 28], without specifying the actual number of patient presenting the abnormality. On average, the latency was 40 ± 4.6% longer than in control patients [15, 27, 28, 18]. In summary, studies (which all involved genetically confirmed FRDA patients, with the exception of [26]) consistently described a frequent pattern of moderately increased latencies [15, 18, 26, 27, 28]. Individuals who showed longer saccade latencies also displayed a higher degree of variability in their latency measurements in one study (Pearson *R* = 0.84, *p* < 0.001) [15]. Furthermore, a correlation was found between the average latency of each individual and their total FARS score (Pearson *R* = 0.66, *p* < 0.05) [15].

##### Saccade Accuracy in Visually-Guided Saccades

Studies reporting on saccade accuracy involved genetically confirmed FRDA patients [15, 18, 27, 28]. 76% of FRDA patients presented with impaired saccade accuracy (71/93 patients from 8 studies). Hypermetric saccades were more frequently observed (19/38) than hypometric saccades (15/38) in 3 studies [18, 19, 26]. In one study where the prevalence of dysmetric saccades in FRDA was compared, hypermetric saccades were found to be more frequent than hypometric saccades, but no statistical analysis was undertaken [15]. Another study reported significantly reduced saccade gain values (gain = 0.87 ± 0.08, *P* < 0.05), i.e. hypometric saccades [28], while two other studies observed normal gain values [19, 27]. Concerning the level of abnormality of saccade accuracy (as assessed by differences in saccadic gain) among the three studies reporting gain values ([19, 27, 28]), two of them did not identify significantly different gain values compared to those from the control groups [19, 27]. This may be due to the presence of both hypermetric and hypometric saccades in the same individual. Noteworthy, centrifugal saccades were found to be hypometric more frequently, while centripetal saccades tended to display overshoot (i.e. being hypermetric) more often [18, 21].

Several factors could have contributed to the broad range of saccade metric abnormalities observed. Firstly, the number of patients included in these studies was relatively small (14 ± 5 [mean ± 1SD]) and they may not have captured the full spectrum of saccadic accuracy in FRDA [15, 18, 19, 26–28]. Second, due to the observed impact of the direction of saccades (centripetal vs. centrifugal) on their metrics, the paradigms applied in those studies included need closer evaluation. Noteworthy, in studies [27] and [28] only centrifugal saccades were recorded. Importantly, when assessing saccade metrics, the direction of saccades relative to straight-ahead gaze needs to be controlled for. Another potential source of variability in reported outcomes lies in methodological distinctions in recording the saccades, specifically regarding saccadic amplitudes. Studies that did not observe hypermetric saccades employed saccadic recording paradigms with amplitude values of 5–20° [27], 10° [28]. In contrast, investigations reporting a higher incidence of hypermetric saccades used on average wider saccadic amplitudes, spanning between 3–36° [26], 40° [19], and 20 or 40° [18].

Moreover, divergent findings in saccade metrics among individuals with FRDA may be attributed to temporal dynamics in the disease progression. Notably, studies [27] and [28], reporting normal or reduced mean gain values, featured cohorts with a comparatively advanced mean age (40 ± 9 years and 35.7 ± 9 years, respectively) and a prolonged mean disease duration (21.3 ± 4 years and 20.5 ± 8.7 years, respectively). Conversely, studies, which reported a higher prevalence of hypermetric saccades, comprised patients with a younger mean age (21 ± 10 years, 30.4 ± 6.5 years, and 39.3, respectively) [18, 19, 26, 32]. Additionally, the mean disease duration in [19] (also reporting a higher frequency of hypermetric saccades) was 15.8 ± 8.3 years. These differences in participant demographics may suggest a potential temporal evolution in saccadic dysfunction. Thus, disease progression may result in a tendency toward saccade hypometria and/or a diminished prevalence of hypermetric manifestations.

##### Saccade Velocity in Visually-Guided Saccades

Saccade velocity was within normal limits in 37/43 FRDA patients assessed in five studies [15, 20, 22, 26, 27]. Reduced saccade velocities were reported in a minority of patients only (6/43) [18, 19, 22, 23, 27]. While one study found a significantly reduced peak velocity compared to the control group (210.64 ± 25.69 vs. 237.00 ± 12.71 deg/s, *p* < 0.01) [28], two other studies did not describe any significant differences in saccade velocity [15, 20]. Moreover, for 30° horizontal saccades, the mean velocity (no SDs reported) in the non-ambulant group (352°/s) was significantly reduced compared to the ambulant group (411°/s; *p* = 0.04) and controls (382°/s; *p* < 0.001) [15]. This might suggest that saccade velocity is initially preserved. Saccade amplitude is another potential influencing factor in velocity-related findings. Studies that did not report individuals with reduced velocities, on average, used saccades of smaller amplitude (3–36° [20, 26], 5–20° [27], and 30° [22]). In contrast, studies that did report instances of reduced velocities employed saccades with relatively larger amplitudes (30° [15], 20 and 40° [18], 40° [19].

##### Anti-Saccades (AS) and Memory-Guided Saccades (MGS)

Those studies reporting on AS and MGS restricted data collection on the horizonal plane, thus no conclusions can be made on plane-specific changes of AS and MGS in FRDA. Compared to VGS, latency and accuracy for AS and MGS appear to be more severely impaired in one study reporting on genetically confirmed FRAD patients [28]. AS latency was increased by 50% (413.4 ± 86.8 ms, two-way ANOVA, *p* < 0.001), while AS absolute position error was augmented by 42% (23.1 ± 11.7%, two-way ANOVA, *p* < 0.05) compared to controls [28]. At the same time, MGS-latency showed a 74% increase (457.1 ± 92.5 ms, two-way ANOVA, *p* < 0.001), while MGS absolute position error appeared to be augmented by 9% (13.9 ± 5.7%, two-way ANOVA, *p* = 0.01) compared to controls [28]. These findings are consistent with the disruption of higher order cognitive control processes governing movement in FRDA patients. The presence of a cognitive component was also suggested by increased latencies in tasks including reprogramming of saccades [32] and by decreased latencies in tasks with attentional disengagement [33].

##### Saccadic Intrusions

Saccadic intrusions (SI) were present in in 89% (143/161 patients from 12 studies) of patients studied. SI occurred during primary gaze, eccentric gaze-holding, PEM and saccades to target. Various types of SI were observed, including square-wave jerks (SWJ) and ocular flutter (OF). SWJ were observed in 83% (92/111) of cases. The mean SWJ-amplitude in the pooled studies was 2.25 ± 0.2° [15, 27] and ranged between 0.5° and 18° [19, 20, 24] while the duration averaged at 207 ± 22.1 ms [15, 27] and ranged between 80 and 540 ms [20, 22, 24]. The duration and size of SWJ were significantly larger (*p* < 0.001) in the absence of a target stimulus (dark/target-off condition, mean = 192 ms) compared to those generated in the presence of a visual target (target-on condition, mean = 158 ms) [15]. Similarly, the mean amplitude was 2.1° in the dark condition, which was significantly higher than 1.8° in the target-on condition (*p* < 0.001) [15]. The total number of SWJ target-on per minute did not show a significant difference between ambulant and non-ambulant patients (ambulant = 298/min, non-ambulant = 327/min; one-way ANOVA, *p* = 0.10) [15]. However, when considering SWJ within the range of 3–30° (macro-SWJ), there was a notable increase in frequency among non-ambulant participants (average for ambulant individuals = 7.4/min, average for non-ambulant individuals = 45.6/min; *p* = 0.02) [15]. It is important to mention that macro-SWJ were overall rarely seen, and 99% of all SWJ observed had amplitudes of less than or equal to 10° [15]. Depending on their degree of amplitude, as well as on the target condition, various correlations were calculated for SWJ (see paragraph “correlations”) [15].

Information on ocular flutter was limited to its presence in 49% of patients (21/43 patients from 3 studies) during straight-ahead gaze and that data on OF came from patients without a genetically verified diagnosis [19, 22, 26].

#### Gaze Holding (Primary/Eccentric Gaze)

Spontaneous nystagmus (SN) was searched in five studies and was present in 10/70 individuals (14%). The SN pattern found was downbeat nystagmus (DBN) in 9/10 cases (all genetically confirmed FRDA patients), all coming from a single study [15]. A possible explanation can be found in the methodology of this publication [15], as it is the only study that provided recordings of spontaneous nystagmus in the vision-denied state, whereas in the other studies [18, 19, 23, 26] vision was permitted. Additionally, periodic alternating nystagmus was reported in one case [18].

If we consider only those studies that measured vertical eye movements, the percentage of SN in FRDA increased to 17% (9/52) [15, 19, 26]. DBN in the vision-denied state correlated with disease duration (Spearman rho = 0.59; *p* = 0.012) [15]. Lastly, in the single study reporting on positional nystagmus in FRDA, no positional nystagmus was found in five patients studied [23].

Horizontal gaze-evoked nystagmus (GEN) was also a frequent finding (38% (40/104 of patients, 8 studies), whereas rebound nystagmus was less often present (14% (10/69) of patients, 5 studies).

#### Optokinetic Nystagmus (OKN)

A minority of patients (34% (23/68), from 7 studies) showed abnormalities of OKN gain [19, 26] or OKN slow-phase velocity [21–27]. One study, employing OKN stimulus velocities of both 30°/sec and 90°/sec, reported a greater percentage of impairment (6 out of 7 patients) [18] than the other studies, all of whom employed an OKN stimulus of not greater than 70°/sec (see Table 1). Only one study reporting about OKN had genetically confirmed patients [18].

### Vestibulo-Ocular Reflex Findings

#### Low-Frequency aVOR, aVOR-Suppression and Response to caloric Irrigation

aVOR abnormalities, such as reduced gain and/or time constant, and increased phase lead, were the most frequently identified vestibular parameter identified in our review, being present in 81% (39/48, 5 studies) of FRDA patients studied. As reported in two studies, the increase of phase lead constitutes a prevalent aVOR abnormality, being present in 23 patients out of 24 studied [26, 30]. Additionally, an abnormal time constant was measured in 8/12 patients in a single study [19]. Conversely, abnormal low frequency aVOR gain, while presenting relatively uncommonly (17/43 patients, 5 studies), has been the subject of greater research enquiry than aVOR time constant or phase lead. The findings in this regard reveal a discernible mean reduction of 44% in gain among the FRDA patients studied [20, 26, 30].

Furthermore, impairments in aVOR-suppression (32/61 patients in 5 studies) and response to caloric irrigation (15/37 patients in 4 studies reporting on patients with non-genetically confirmed FRDA) were not uncommon among FRDA patients. aVOR-suppression gain was augmented by 120% in patients from three studies [18, 20, 26]. Concerning the response to caloric irrigation, one study reported a mean value of the slow-phase velocity of the generated nystagmus equal to 77 ± 54°/sec, compared to 117 ± 35°/sec in a healthy control group, reflecting a 44% reduction in response magnitude [19]. In the other studies [19, 21–27], at least eight patients out of 37 exhibited a maximal slow-phase velocity response of less than 5°/sec. Caloric responses were generally symmetrical [21, 22] and always bilaterally reduced in a single study [23].

#### Video Head-Impulse Testing (vHIT)

The horizontal vHIT-gain was reduced by approximately 50% in the patients compared to the controls in two studies reporting on genetically confirmed FRDA patients (0.48 ± 0.20 [mean ± 1SD] vs. 1 [SD not reported] [15]; 0.42 ± 0.17 vs. 0.94 ± 0.08 [31]) and the vHIT-latency appeared to be significantly increased in patients compared to controls in both studies (26 ± 0.5 ms vs. <10ms [15]; 23.7 ± 15.7 ms vs. -2.3 ± 2.1 ms (*p* ≤ 0.001) [31]). The vHIT allows for the assessment of compensatory saccades generated both during the head impulse (covert saccades) and following the head impulse (overt saccades). A single study looked for these catch-up saccades and found that overt saccades were present in almost all patients (8/9), with the absence of covert saccades, possibly related to lacking adaptational mechanisms [31].

### Correlations Between Oculomotor / Vestibulo-Ocular Reflex Parameters and Other Abnormalities in FRDA

This systematic review also aimed to examine any associations between oculomotor / vestibulo-ocular reflex abnormalities and other relevant parameters. The range of correlations identified included age at symptom-onset, disease duration, and clinical scale scores (FARS, SLCLC and SARA). In considering correlations with disease stage or duration, data from four studies was identified. In Tables 5, 6 and 7 we incorporated the Pearson and Spearman correlation values obtained from the respective studies, except for values from [27], which were calculated by our team using data extracted from the study. We only identified one study with longitudinal quantitative oculomotor / vestibulo-ocular reflex data [29]. In the four studies, correlations were calculated using information on disease duration, age at symptom onset and severity scores contained in the publications [15, 28, 31, 32].

**Table 5:** Correlations: SWJ frequency (Pearson R values)

	SWJ	SWJ target on	SWJ target on > 3°	SWJ target off	SWJ target off 0°-0.5°	SWJ target off > 3°	SWJ duration
Age at symptom-onset	-0.46 [27]	-0.57* [15]	-0.55* [15]	-0.58* [15]		-0.63* [15]	0.83** [15]
Disease duration	-0.27 [27]	0.33 [15]	0.56* [15]	-0.04 [15]		0.45 [15]	
Longer GAA repeat length		0.27 [15]	0.08 [15]	0.48 [15]	0.64** [15]	0.23 [15]	
FARS		0.52 [15]	0.68* [15]	0.17 [15]		0.60 [15]	
SARA	0.3 [27]						
SLCLC		-0.55* [15]	-0.82* [15]	-0.61* [15]		**-0.73** [15]	

Values in bold are significant

*P ≤ 0.05, **P ≤ 0.01

*Abbreviations* FARS = Friedrich’s Ataxia Rating Scale; SLCLC = Sloan Low Contrast Letter Chart; SARA = Scale for the Assessment and Rating of Ataxia; SWJ = square-wave jerk

**Table 6:** Correlations: saccadic eye movements (Pearson R values)

	VGS gain	VGS velocity	VGS horizontal latency	VGS latency	AS latency	Diff. VGS/AS latency	MGS errors
Age at symptom-onset	-0.44 [27]	-0.27 [27]	-0.3 [27]	-0.34 [28]	-0.45 [28]	-0.22 [28]	0.21 [28]
Disease duration	-0.22 [27]	-0.11 [27]	0.26 [27]	0.41 [28]	0.64* [28]	0.12 [28]	0.77** [28]
Smaller GAA repeat				0.04 [28]	-0.17 [28]	-0.03 [28]	-0.66* [28]
FARS	**-**	**-**	0.66* [15]	0.77** [28]	0.75** [28]	0.20 [28]	
SARA	0.33 [27]	-0.39 [27]	0.75* [27]	-	-	-	
SLCLC	**-**	**-**	-0.78* [15]	-0.82** [28]	-0.78** [28]	-0.55 [28]	

Values in bold are significant

*P ≤ 0.05, **P ≤ 0.01

*Abbreviations* AS = anti-saccades; diff = difference; FARS = Friedrich’s Ataxia Rating Scale; MGS = memory-guided saccades; SLCLC = Sloan Low Contrast Letter Chart; SARA = Scale for the Assessment and Rating of Ataxia; SWJ = square-wave jerk; VGS = visually-guided saccades

**Table 7:** Correlations: DBN and aVOR (Pearson R values)

	DBN	aVOR gain (vHIT)	aVOR peak gain (vHIT)	aVOR peak latency (vHIT)
Age at symptom onset	-	-	-	-
Disease duration	0.59*† [15]	-	-	-
FARS	-	-	-0.34 [15]	0.63* [15]
SARA	-	-0.46**§ [31]	-	-
SLCLC	-	-	0.59* [15]	-0.41 [15]

Values in bold are significant

* *p* ≤ 0.05, ** *p* ≤ 0.01

† = Spearman rho value

§ Note that a mixed patient cohort consisting of both FRDA patients (*n* = 9) and patients with various spinocerebellar ataxias (SCAs, *n* = 24) was used and that no data on disease-specific subgroups was provided

*Abbreviations* aVOR = angular vestibulo-ocular reflex; DBN = downbeat nystagmus; FARS = Friedrich’s Ataxia Rating Scale; SLCLC = Sloan Low Contrast Letter Chart; SARA = Scale for the Assessment and Rating of Ataxia; SWJ = square-wave jerk; vHIT = video-head-impulse test

In one study, the frequency of SWJ in different testing conditions (see Table 5) demonstrated a negative correlation with the age at symptom onset, whereas SWJ duration showed a positive correlation (*R* = 0.83, *p* ≤ 0.01) [15], that is, individuals who developed the disease early presented with a significantly higher frequency and shorter duration of SWJ [15]. The correlation between age at symptom onset and SWJ was absent in a second study (however, no values were reported, *p* > 0.01) [26] and in a third did not reach statistical significance but showed only a discernible negative trend (see Table 5) [27]. A clear negative correlation was observed between SWJ and the SLCLC (see Table 5) in one study [15]. Only macro-SWJ (i.e., size > 3°) showed a significant correlation with disease duration and the FARS [15]. Moreover, [29] points out that “All patients undergoing testing had SWJ at the end of the study, compared with 35 of 37 at the beginning”, and that “The frequency of SWJ increased (…) at the end of the study”, with a study duration ranging from six months to seven years (median period = 5 years). Interestingly, [21] asserts that SWJ were more often observed in patients having a severely deranged pursuit system. Despite these results, other studies did fail to identify any significant correlation between the disease duration and SWJ [19, 26]. The only longitudinal study available did not yield conclusive findings about the evolution of SWJ in untreated patients (with oculomotor data available in only 3 out of 16 patients studied) due to a lack of data. This study, however, was able to find a significant increase in SWJ frequency per year (0.09 ± 0.02 Hz; *p* < 0.001) in patients treated with idebenone (*n* = 88), a coenzyme Q10 analogue [29].

Latency of horizontal VGS demonstrated a significant positive and negative correlation, respectively, with the FARS [15] and the SLCLC [15, 32] (see Table 6). This is the only parameter for which correlation values were calculated in the two studies. Similarly, VGS latency positively correlated with the SARA in another study [27]. For AS latency the correlations with the severity scores were even stronger, and a positive correlation with disease duration was also found [28].

While DBN correlated with disease duration [15], aVOR gain, aVOR peak gain and aVOR latency (all in vHIT studies) appear to correlate with FRDA severity scores [15, 31] (see Table 7). aVOR peak gain positively correlated with the SLCLC (Pearson *R* = 0.59, *p* ≤ 0.05) and aVOR peak latency positively correlated with the FARS (Pearson *R* = 0.63, *p* ≤ 0.05) [15]. However, in another study, a negative correlation with aVOR gain and disease severity, as evaluated by the SARA (Spearman rho = − 0.46, *p* = 0.01) was found [31]. In this case, however, the patient group included both FRDA patients (*n* = 9) and patients with different types of spinocerebellar ataxia (SCA; *n* = 23) and no correlation analyses were provided for single disease entities.

Concerning correlations with genetic parameters, larger GAA repeat expansions demonstrated a significant correlation with the frequency of SWJ in the dark condition and having an amplitude between 0° and 0.5° (Pearson *R* = 0.64, *p* ≤ 0.01) [15]. Shorter GAA repeat length was significantly and negatively correlated with MGS errors (Pearson *R* = -0.66, *p* ≤ 0.05) [28]. Despite the absence of significant correlations between GAA repeat length and severity scores such as FARS or SLCLC [15, 28], a significant correlation was observed in one study between the extent of the GAA expansion and the duration from disease onset to wheelchair use [29]. Patients with GAA repeat length exceeding 2 kb required a wheelchair significantly earlier than their counterparts, showing a relative risk of 2.86 (95% confidence interval = 1.76–4.63) [29].

## Discussion

The primary aim of this systematic review and meta-analysis was to provide a comprehensive overview of oculomotor and vestibulo-ocular reflex abnormalities observed in FRDA patients by use of quantitative measurements, and to highlight those abnormalities most suitable as for facilitating early detection and diagnosis as well as the monitoring of disease progression in natural history studies and symptom improvement in treatment trials. The most frequent abnormalities observed in FRDA patients were saccadic intrusions (SI) (89%), altered pursuit eye movements (PEM) (87%) and angular vestibulo-ocular reflex (aVOR) impairments (81%). If we consider studies involving patients with a genetically confirmed diagnosis, the frequency fractions are even higher (96% for PEM [15, 18], 93% for SI [15, 27, 18, 29] and 91% for SWJ [18, 27, 29]. Pursuit gain reductions were frequently reported but typically mild [18–20, 26], matching the observation made in most hereditary ataxias, with the exceptions of SCA3 and SCA6, presenting frequently reduced pursuit velocity and prominent gain reduction, respectively [11].

Dysfunction of the superior colliculus-omnipausal neuron pathway (producing a bilateral tonic inhibition to the pontine paramedian reticular formation [PPRF] via the omnipause neurons) has been proposed to explain alterations in saccadic eye movements, SWJ and gaze-holding in FRDA [15, 34]. With projections to the superior colliculus, both cerebellar pathways and the frontal and parietal eye fields may thus affect eye movements in FRDA patients [35]. At the same time a significant reduction in volume and white matter degeneration in the superior cerebellar peduncle has been demonstrated on brain imaging in FRDA patients [36–38], providing a structural correlate for disrupted output from the cerebellum [33].

Disease progression may result in a tendency toward saccade hypometria and/or a diminished prevalence of hypermetric manifestations. With regards to the presence of both hypometric and hypermetric saccades in single patients, Fahey and colleagues have proposed that underlying cerebellar pathology may generate saccadic instability. This hypothesis was based on lesion models and imaging. Specifically, models of saccadic generation have indicated a cerebellar role in saccadic accuracy by modulating burst neuron function [39]. Furthermore, increased iron-deposits in the dentate nucleus have been demonstrated in FRDA patients, indicating cerebellar dysfunction [40]. Concerning other hereditary ataxias, the reduction in saccade velocity may surpass that observed in FRDA. This includes SCA1, SCA2 [11, 15] SCA7, SCA8 [15] and Niemann-Pick disease type C [11].

Overall, the range of oculomotor and aVOR abnormalities observed in FRDA patients emphasizes a combined peripheral and central vestibular impairment. Whereas the presence of DBN and rebound nystagmus, aVOR-suppression impairment, and SI point to central (cerebellar) involvement, the reduced responses to caloric irrigation and lowered vHIT-gains suggest peripheral lesions. This reflects known temporal bone histopathological findings demonstrating significant spiral ganglion cell loss with a near normal organ of Corti [41, 42]. Additionally, histopathological abnormalities in Friedreich ataxia includes vestibular (Scarpa’s) ganglia cell loss with secondary vestibular nerve atrophy [43]. Interestingly, the vestibular end organs have been found to be histopathologically unaffected.

### Proposed Oculomotor / Vestibulo-Ocular Reflex Paradigms in FRDA Patients

Utilising the results of the present work, a fingerprint of oculomotor and aVOR deficits in FRDA can be constructed (see Table 8 for details). While a broad range of paradigms has shown abnormalities in FRDA patients, the frequency and the magnitude of the abnormalities varied (see also Table 1). Thus, focusing on the paradigms that have been associated with frequent and pronounced alteration in FRDA patients is recommended. Such a “core” set of oculomotor and aVOR paradigms should include an assessment saccadic eye movements (saccadic intrusions, increased saccadic latency and decreased saccadic accuracy) and aVOR-responses (gain reduction) assessed by caloric irrigation or vHIT. Specific parameters for measuring these domains are straight-forward. Saccadic intrusions are best caught during gaze straight-ahead (for at least 60 s), visually-guided saccades should be applied using reliable velocities in the range of 10–30° in the horizontal plane), and for both caloric irrigation [44–46] and video head-impulse testing [46, 47] paradigms and normative values have been provided.

**Table 8:** FRDA oculomotor / vestibulo-ocular reflex “fingerprint”

Domain	Key oculomotor / vestibulo-ocular reflex changes
Gaze-holding (eccentric gaze)	• Impaired eccentric gaze holding (only considering GEN)** in a significant fraction [15, 18–21, 23, 24, 26]** • Rarely rebound nystagmus [18, 19, 21–23, 26]**
Optokinetic nystagmus	• Sometimes reduced gain [19, 26]* or/and slow phase velocity [21–23]*
Pursuit eye movements	• Frequent SI [15, 20–26]**and catch-up saccades [15, 21]* • Mildly reduced gain [18–20, 26]**
Saccadic eye movements	
Saccadic intrusions	• Frequent SI with SWJ** [18–21, 24, 26, 29] and OF* [19, 22, 26] • Rarely macro-SWJ*** [15]
Anti-saccades and memory-guided saccades	• Strongly augmented latency [28]*** • Moderately reduced AS accuracy [28]*** • Mildly reduced MGS accuracy [28]***
Visually-guided saccades	• Frequent increases in saccadic latency [18, 26] **of moderate extent [15, 18, 27, 28]*** • Frequent dysmetria [18–23, 25, 26]** • Rarely reduced velocity [18, 19, 22, 23, 27]**
Spontaneous nystagmus	• Rarely DBN [15, 18, 19, 23, 26] §
Angular vestibulo-ocular reflex (VOR)	
Caloric irrigation	• aVOR-gain reduced in a significant fraction [19, 21–23]*
High-frequency aVOR (vHIT)	• Moderately to strongly reduced aVOR-gain [15, 31]*** • Strongly augmented aVOR-latency [15, 31]*** • Presence of overt catch-up saccades [31]*** • Lack of covert catch-up saccades [31]***
Low-frequency aVOR	• Frequently impaired [18, 19, 23, 26, 30]** • Frequent increases in phase lead [26, 30]** • Time constant impaired in a significant fraction [18, 19, 26, 30]** • Sometimes impaired gain [18, 19, 26, 30]**
aVOR suppression	• Impaired in a significant fraction [18, 19, 21, 22, 26]**

* This oculomotor / vestibular finding was studied only in patients with non-genetically confirmed FRDA

** This oculomotor / vestibular finding was observed in both patients with genetically confirmed FRDA, and diagnosis based on clinical presentation or family history

*** This oculomotor / vestibular finding was studied only in patients with genetically confirmed FRDA

§ This oculomotor finding was studied in both patients with genetically confirmed FRDA, and diagnosis based on clinical presentation or family history, but all the patients showing this abnormality were genetically confirmed

*Abbreviations* AS = anti-saccades; aVOR = angular vestibulo-ocular reflex; DBN = downbeat nystagmus; GEN = gaze-evoked nystagmus; MGS = memory-guided saccades; OF = ocular flutter; SI = saccadic intrusions; SWJ = square-wave jerks; vHIT = video-head-impulse test

A prominent prevalence of SWJ is also identified in spinocerebellar ataxia (SCA) type 6 (80–100%), SCA3 (43–64%), ataxia-telangiectasia (31–85%) [11], SCAR4 (formerly SCASI and SCA24) [48], pointing to relevant differential diagnoses. In contrast, SCA1 and SCA2 patients less often display SWJ [11] (20–30%). While SWJ represent a non-specific parameter, OF, in the context of hereditary ataxias, has been observed exclusively in FRDA and ataxia-telangiectasia [11]. On the other hand, DBN was an infrequent finding in publications included in this review and is known to be observed in a range of other hereditary ataxias, including SCA3, SCA6, SCA17, SCA27B, Niemann-Pick disease type C, and CANVAS/RFC-1 related disorders [11, 49]. Thus, its differentiating value for FRDA diagnosis is limited. Furthermore, in FRDA moderately increased saccadic latencies were observed. In general, augmented saccadic latency seems to be a rather common feature in hereditary ataxias although in SCA3 saccadic latency seems to be relatively preserved [11]. Thus, the value of saccade latency as discriminatory finding from other hereditary ataxias is limited.

Given the sensitivity and specificity, as well as the ease of access and use of the vHIT, it might well be valuable in the differential diagnosis of FRDA from SCA1, SCA2 and SCA3 particularly when considering the distinct patterns of vHIT abnormalities in these diseases. Firstly, the vHIT-gain has been found to be normal in SCA1 and only slightly reduced in SCA2 [31]. Secondly, aVOR-latency is preserved in SCA1 and SCA2 whilst lastly, covert saccades were only observed in SCA3 [31].

### Early Disease Detection, Measurement of Disease Progression and Disease Severity

Examining the associations between oculomotor / aVOR abnormalities and the stage or duration of disease was a key objective of this systematic review (see Table 9 for summary). First, our data revealed a remarkable negative correlation between the SWJ frequency and the age at symptom-onset, particularly pronounced in instances of macro-SWJ in darkness [15]. However, the correlation between the SWJ frequency and the age at symptom-onset, could not be confirmed in other studies [26, 27]. Additionally, a robust correlation exists between the duration of SWJ and age at symptom onset [15]. Furthermore, macro-SWJ (target-on) exhibited a positive correlation with disease duration and the FARS, concomitant with a negative correlation with the SLCLC [15]. This suggests a potential dichotomy where shorter SWJ duration may serve as indicative marker for early disease detection in young patients, while macro-SWJ (target-on) may be a marker for disease severity. A clear negative correlation was found between SWJ frequency and the SLCLC (see Table 5), while, as just stated, only macro-SWJ (target-on) positively correlated with the FARS [15]. In general, the link between SWJ frequency and diseases duration was not significant [19, 26, 27].

**Table 9:** The value of oculomotor / vestibulo-ocular reflex parameters as biomarkers in FRDA*

Clinical assessment	Parameters
Potential markers for early onset FRDA	• Frequent short SWJ [15]
Potential markers for assessing disease duration	• Augmented AS latency [28] • DBN [15] • Frequent macro-SWJ (target-on condition) [15]
Potential markers for quantifying disease severity and monitoring disease progression	• Augmented AS latency [28] • Augmented VGS latency [27] • Frequent macro-SWJ (target-on condition) [15] • Reduced aVOR gain and aVOR peak gain (vHIT) [15, 31] • Augmented aVOR peak latency (vHIT) [15]

* Note that all studies referred to in this table included only patients with genetically confirmed FRDA

*Abbreviations* AS = anti-saccades; aVOR = angular vestibulo-ocular reflex; DBN = downbeat nystagmus; FRDA = Friedreich Ataxia; MGS = memory-guided saccades; SWJ = square-wave jerks; VGS = visually-guided saccades; vHIT = video-head-impulse test

The significant positive correlations between (horizontal) VGS latency and the FARS [15, 28] alongside the negative correlation with the SLCLC [15, 28] underscore the potential clinical relevance of this parameter. The fact that these correlations were consistently observed across two independent studies enhances the robustness and reliability of these associations. Additionally, the positive correlation observed between VGS latency and the SARA [27] suggests that increased VGS latency is indicative of greater disease severity.

Anti-saccade latency was positively correlated with the FARS score and disease duration, as well as negatively correlated to the SLCLC. [28]. This emphasizes the potential utility of AS latency as a sensitive marker for overall disease progression. Additionally, these findings imply that with disease progression, there is an observable decline in higher cognitive function. In fact, a recent systematic review confirms the correlation between disease severity and cerebellar structural parameters, and neurocognitive deficits in a range of domains (including language, attention, executive, memory, visuospatial perceptual, emotion recognition and social cognitive abilities) [50].

The correlation between DBN and disease duration suggests that this oculomotor abnormality may manifest and progress over the course of disease and reflect the increasing cerebellar degeneration [15]. The correlations observed between vHIT parameters (aVOR gain, aVOR peak gain, and aVOR latency) and various FRDA severity scores (FARS, SARA, SLCLC) [15, 31] indicate that these parameters may offer material insights into various facets of disease severity (see Table 6). A notable correlation is observed between aVOR peak gain and the SLCLC, suggesting that diminished peak gain may be associated with contrast vision disturbances and impairments in visual acuity [15]. Additionally, the aVOR peak latency correlates with the FARS score [15], indicating that augmented latency may be linked to motoric disturbances. In summary, these findings imply that alterations in aVOR parameters could provide insights into specific clinical manifestations, with reduced peak gain potentially relating to visual factors whilst reduced latency is potentially instructive in reflecting more general motoric manifestations of ataxia. There may be a negative correlation between the aVOR gain and disease severity (as assessed by the SARA), but since data from patients with not only FRDA but also SCA1, 2 and 3 were considered to calculate the correlation, this requires further investigation [31].

Lastly, the results concerning correlations between oculomotor parameter or severity scores and GAA repeat length suggest that genetics may have complex associations with the clinical presentation of FRDA. Longer GAA repeat lengths appear to correlate with SWJ in the dark condition having an amplitude between 0° and 0.5° [15] and the shorter GAA repeat length has significantly and negatively correlated with MGS errors [28]. Moreover, patients with GAA repeat length exceeding 2 kb required a wheelchair significantly earlier [29]. If, on one hand, this could imply implying an association with disease severity, on the other, no significant correlation was found between the repeat length and severity scores or other oculomotor / vestibulo-ocular reflex parameters [15, 28], suggesting that other factors might also be involved in determining the disease phenotype of FRDA.

### Limitations

A limitation of this systematic review is the variability in measurement methods across studies. This was present at several levels: different measuring equipment, varying numbers of plains measured (horizontal or horizontal and vertical) and discordance in the specific conditions such as stimulus velocity or saccade amplitudes. Given this heterogeneity, a meta-analysis across studies was often not possible, limiting the power of certain parameters identified to single studies. This was particularly the case for OKN and saccadic velocity. Furthermore, definitions of eye movement types recorded were sometimes vague or even lacking (as e.g. for ocular flutter or SWJ), possibly resulting in wrongly classified eye movement recordings. As previously enumerated, another limitation is the insufficient patient data included in certain studies. The almost complete absence of longitudinal studies functions to limit the veracity of any correlations that may be drawn with clinical changes over time. These factors underscore the need for standardized data collection methods and longitudinal observational studies to better understand FRDA and its associated eye movement abnormalities.

## Conclusions

To our knowledge, this study represents the first systematic review / meta-analysis on quantitative oculomotor / vestibulo-ocular reflex parameters in FRDA. The most frequently observed eye movement abnormalities in FRDA patients were the presence of SI (89%), mostly SWJ (83%), and deficits in PEM (89%). Other common changes were increased VGS latency (71%) and reduced VGS accuracy (76%). aVOR abnormalities were another important finding, including aVOR-gain reduction with caloric irrigation (41%), phase-lead increase (96%) and time constant impairment (66%) in low-frequency aVOR, as well as aVOR suppression impairment (52%). In high-frequency head-impulse testing we noted significantly increased aVOR latencies and decreased aVOR gains. SWJ amplitude, AS and VGS latency, DBN frequency, aVOR gain and latency showed correlations with disease onset, duration or severity. Thus, measuring PEM, SEM (including SI) and aVOR responses should be prioritized as metrics of disease severity. Our finding that disease progression may result in a tendency toward saccade hypometria and/or a diminished prevalence of hypermetric manifestations has not been further investigated to our knowledge and therefore presents a potentially valuable avenue of future endeavor. In summary, quantitative oculomotor testing in FRDA may facilitate early diagnosis and provide value in monitoring disease progression and treatment response. Future research in the field should prioritize standardized data collection methods and longitudinal observational studies to better understand FRDA and its associated eye movement abnormalities.

## Electronic Supplementary Material

Below is the link to the electronic supplementary material.

Supplementary Material 1

Supplementary Material 2

## Data Availability

The data that support the findings of this study are available from the corresponding author upon reasonable request.
