# Supplementary material for: Oculomotor and Vestibular Deficits in Friedreich Ataxia - Systematic Review and Meta-Analysis of Quantitative Measurements
Source: Cerebellum. 2024 Jul 27;23(6):2269–84. doi: 10.1007/s12311-024-01716-8 (PMC11585506; doi:10.1007/s12311-024-01716-8)
Supplement: Supplementary file 1 — Supplementary Material 1 [file 12311_2024_1716_MOESM1_ESM.docx]

## Appendix 1: Electronic search strategy, coding-scheme for the systematic review and data analysis

**The search strategy was designed by a clinical investigator with relevant domain expertise in neurology (AAT).**

**We searched MEDLINE and Embase for English-language articles, using the following strategies with the following components: (1) defining the clinical syndrome (i.e., ataxia), (2)** ocular motor or vestibular features**, and (3) quantitative assessments. We did not expressly search for hereditary ataxia syndromes, as this was found to result in omitting most relevant studies due to lacking referral to the genetic background. We also performed a manual search of reference lists from eligible articles, and contacted corresponding authors where necessary. We did not seek to identify research abstracts from meeting proceedings or unpublished studies.**

MEDLINE Search *(accessed via PubMed at www.ncbi.nlm.nih.gov/pubmed)*

(Friedreich ataxia OR FRDA) AND ((eye movements) OR (ocular motor) OR oculomotor OR vestibular OR saccade OR (smooth pursuit) OR (vestibulo-ocular reflex) OR VOR OR optokinetic OR nystagmus OR gaze OR head impulse OR caloric) AND (quantitative OR recording OR recordings OR quantified OR measured) NOT (animals[mh] NOT humans[mh]) AND eng[la] NOT review[pt])

Embase search

(‘Friedreich ataxia’:ab,ti OR FRDA:ab,ti) AND (‘eye movements’:ab,ti OR ‘ocular motor’:ab,ti OR oculomotor:ab,ti OR vestibular:ab,ti OR saccade OR ‘smooth pursuit’:ab,ti OR ‘vestibulo-ocular reflex’:ab,ti OR VOR:ab,ti OR optokinetic:ab,ti OR nystagmus OR gaze OR ‘head impulse’:ab,ti OR caloric*:ab,ti) AND (quantitative:ab,ti OR recording*:ab,ti OR quantified:ab,ti OR measured:ab,ti) AND [humans]/lim AND [English]/lim AND ([article]/lim OR [article in press]/lim)

Search Results

Our search identified 32 unique citations and 3 additional records were included after an additional literature research based on a recent systematic review on oculomotor assessment in hereditary ataxias from the same group and on a recent systematic review on neuro-ophtalmological findings in FRDA. Thus, of 35 papers screened, 10 (29%) were excluded at the abstract level (Figure 1, main manuscript). A record was excluded only if two scorers (ES, AAT) recommended exclusion (detailed list of predefined reasons for exclusion shown below). We did not demand concordance on reason for abstract exclusion, but, among those abstracts with concordant reasons for exclusion (29%, n=10), the distribution was as follows: **6 were not reporting on the assessment of oculomotor /vestibular features**; 4 had no data on human subjects with Friedreich ataxia **and 4 were not reporting on quantitative measurements.**

We further examine 25 full manuscripts. After initial screening, there were no disagreements about study inclusion for the two reviewers (ES and AAT, kappa=1.0]). Overall agreement on reason for exclusion was 57%. We demanded concordance on reason for full-text exclusion and resolved differences by discussion.

At the end of our full-text review, 8 were excluded and 17 were considered eligible (Figure 1, main manuscript). These eligible studies represented 68% of the total (n=25). Among all full-text manuscripts excluded (32%), the distribution of reason for exclusion was as follows: **4 were not reporting on the assessment of oculomotor /vestibular features**; **3 were not reporting on quantitative measurements and** 1 had no data on human subjects with Friedreich ataxia**;**

### Coding schema for abstract and full-text reviews

**All gathered literature was subject to title/abstract screening by two independent reviewers (ES and AAT). Full-text screening was then applied to all citations considered eligible or possibly eligible by at least one reviewer. Two independent reviewers (ES and AAT) determined whether full-text manuscripts were eligible and, if not, provided a reason for exclusion. Differences were resolved by discussion and consensus. AAT completed a hand search of the reference lists of selected articles for additional citations. For citations identified by hand search, the full process was repeated iteratively until no additional manuscripts were found for inclusion. We calculated inter-rater agreement on full-text inclusion using Cohen’s kappa [1]. A formal review protocol was not registered or posted.**

Abstract Review Coding Rules

1) Coding status options are “Yes”, “No”, “Maybe”. We will review full text of “Yes” and “Maybe”. The purpose of “Yes” vs. “Maybe” is to look at kappa values agreement on “Yes” vs. “Maybe”.

2) Err on the side of “Maybe” if there is doubt about a “No”; this is more conservative.

3) If there is only a title, exclude it only if you feel confident; otherwise code it as “Maybe”.

4) Each "No" or "Maybe" should be coded with a reason for exclusion.

5) Reasons for exclusion are listed below 0-5. Go through them in order from 0 to 5 for each abstract, coding the first reason for exclusion only, not multiple reasons for exclusion. Only code "0" for “not English” if you are sure it is “not English”.

6) Two independent raters will code reason for exclusion, but we will not mandate agreement on exclusion reason at the abstract level.

7) Occasionally an abstract seems inappropriate for another reason. In such cases, code as “other”. There should be few “other” codings.

Abstract Reasons for Exclusion

| 0 | not English | manuscript is not in English |
| --- | --- | --- |
| 1 | no data | review paper; no original patient data |
| 2 | not ataxia | No data on human subjects with Friedreich ataxia |
| 3 | not oculomotor / vestibular | not reporting on the assessment of oculomotor and/or vestibular features |
| 4 | not quantitative | not reporting on quantitative oculomotor and/or vestibular measurements |
| 5 | other | any other reason abstract is not included |

Full-Text Review Coding Rules

1) Coding status options are “Yes” or “No”.

2) Each "No" should be coded with a reason for exclusion.

3) Reasons for exclusion are listed below 0-4. Go through them in order from 0 to 4 for each full text, coding the first reason for exclusion only, not multiple reasons for exclusion.

4) Two independent raters will code reason for exclusion, and we will mandate agreement on exclusion reason at the full text level.

5) Coding differences will be adjudicated or consensus will be developed through dialogue.

Full-Text Reasons for Exclusion

| 0 | not English | manuscript is not in English |
| --- | --- | --- |
| 1 | no data | review paper; no original patient data |
| 2 | not ataxia | No data on human subjects with Friedreich ataxia |
| 3 | not oculomotor / vestibular | not reporting on the assessment of oculomotor and/or vestibular features |
| 4 | not quantitative | not reporting on quantitative oculomotor and/or vestibular measurements |

**References**

[1] Cohen J. A coefficient for agreement for nominal scales. Educ Psychol Meas 1960: 20:37-46.
