# Supplementary material for: Oculomotor and Vestibular Deficits in Friedreich Ataxia - Systematic Review and Meta-Analysis of Quantitative Measurements
Source: Cerebellum. 2024 Jul 27;23(6):2269–84. doi: 10.1007/s12311-024-01716-8 (PMC11585506; doi:10.1007/s12311-024-01716-8)
Supplement: Supplementary file 2 — Supplementary Material 2 [file 12311_2024_1716_MOESM2_ESM.docx]

**Appendix 2 - quality assessment for studies reporting on oculomotor findings in FRDA**

Two independent reviewers (ES and AAT) rated all included studies with regards to their quality in reporting oculomotor testing in FRDA patients based on the previously published quality assessment criteria by Garces et al. (REF). Discrepancies were resolved by discussion. Based on the eight items listed below an overall quality rating (high, moderate, low) was assigned. “High quality” was defined as having high quality on items 1, 3-7 and a low risk-of-bias for item 8. Item 2 was not considered since, depending on the study design a control group may not be required (e.g. for treatment response studies). “Moderate quality” studies were defined as studies having at least moderate quality ratings for all three items (with „low“ or „high“ risk of bias for item 8). All studies that received a low-quality rating in one or several items (items 1-7) or had an unknown risk of bias for item 8 were considered “low quality” studies.

**Criteria for the quality assessment of reporting oculomotor findings in FRDA (from [1])**

**Study cohort related items**

1. Patient selection

high = FRDA in all patients included was confirmed by genetic testing

moderate = no genetic testing available but either positive family history with a clear pattern of inheritance autosomal dominant [AD], autosomal recessive [AR], X-linked recessive [XR]) or established and specific diagnostic biomarkers used

low = clinically- or imaging-based diagnosis, no genetic testing, no positive family history

2. Control group selection

high = control group age-matched, independent from test group, clinical assessment (exclusion of abnormal findings) provided in detail; patients serve as their own controls in longitudinal treatment trials or treatment and placebo groups were age-matched in placebo-controlled treatment trials.

moderate = age-matched, reportedly healthy but no details reported how this was assessed

low = one or several of the following: controls are not age-matched; no information about their age is available; no information about selection process is provided; controls are genetically undetermined relatives of the test patients included.

not available = no control group is provided

**Items related to data acquisition**

3. Rigidity of recording protocol applied

high = pre-specified recording protocol available for all parameters studied, identical protocol applied in all participants and sessions

moderate = pre-specified recording protocol available only for selected parameters or different pre-specified recording protocols applied in included patients and sessions.

low = no pre-specified recording protocol used or no information provided about the use of a recording protocol.

4. Description of recording parameters used

high = all recording parameters are reported with sufficient detail to reproduce the study

moderate = recording parameters are reported partially only

low = no information provided about recording parameters

5. Appropriateness of recording devices used for oculomotor testing

high = high temporal and spatial resolution, high signal-to-noise recording technique used (e.g. search coils, high-quality video-oculography with sampling rate of >=100Hz)

moderate = recording technique with moderate to high noise levels (e.g. electro-oculography, electro-nystagmography, low to middle performance video-oculography (sampling rate <100Hz)) or behavioral paradigm used (e.g. Subjective Visual Vertical)

low = no details about recording device reported or high noise levels described

**Items related to data analysis**

6. Description of data analysis

high = data analysis is described with sufficient detail to allow reproduction of study

moderate = data analysis description is provided, but details are lacking and thus is insufficient to allow reproduction of study

low = no information provided about how the data analysis was performed or inadequate analysis pipeline

7. Assessment of obtained test results for significance

high = statistical analysis using normative values obtained from a control group or baseline values (for longitudinal studies)

moderate = statistical analysis using normative values from published literature or from the manufacturer of the device used

low = no normative values available and/or no statistical analysis performed

8. Risk of bias for assessing test results

low = index test results interpreted in a blinded fashion (i.e., without knowledge if the underlying disorder was present or absent) or automated data analysis without rater’s input.

high = index test results interpreted in a non-blinded fashion only and / or based on the rater’s subjective assessment (e.g. rating of overall pattern of vHIT traces).

unclear = no information provided about blinding of reviewers of test results.

**Table S2: quality assessment for studies reporting on oculomotor findings in FRDA – overview on all selected studies**

| Table S2: quality assessment for studies reporting on oculomotor findings in FRDA – overview on all selected studies | | | | | | | | | |
| --- | --- | --- | --- | --- | --- | --- | --- | --- | --- |
| **Study** | **Patient selection** | **Control group selection** | **Rigidity of recording protocol applied** | **Description of recording parameters used** | **Appropriateness of recording devices used for OM /vestibular testing** | **Appropriateness of data analysis applied** | **Assessment of obtained test results for significance** | **Risk of bias for assessing test results** | **OVERALL study quality rating** |
| Alexandre et al. 2013 [2] | high | moderate | high | high | high | moderate | high | low | moderate |
| Baloh et al. 1975 [3] | low | low | high | low | moderate | moderate | low | high | low |
| Ciuffreda et al. 1985 [4] | low | not available | low | moderate | moderate | low | low | high | low |
| Crane et al. 2000 [5] | low | low | high | high | high | high | high | low | low |
| Dale et al. 1978 [6] | moderate | not available | high | moderate | moderate | moderate | low | high | moderate |
| Ell et al. 1984 [7] | moderate | not available | high | moderate | moderate | moderate | low | high | moderate |
| Fahey et al. 2008 [8] | high | low | high | high | high | high | high | low | low |
| Fielding et al. 2010 [9] | high | moderate | high | high | high | high | high | low | high |
| Furman et al. 1983 [10] | low | low | high | high | moderate | low | high | unclear | low |
| Hocking et al. 2014 [11] | high | moderate | high | high | high | high | high | low | high |
| Hocking et al. 2010 [12] | high | moderate | high | high | high | high | high | low | high |
| Kirkham et al. 1979 | low | low | high | high | moderate | moderate | low | unclear | low |
| Luis et al. 2016 [13] | high | high | high | high | high | high | high | low | high |
| Moschner et al. 1994 [14] | low | moderate | high | high | moderate | moderate | high | low | low |
| Ribai et al. 2007 [15] | high | high | high | high | moderate | moderate | high | unclear | low |
| Spieker et al. 1995 [16] | moderate | high | high | high | moderate | high | high | low | moderate |
| Wessel et al. 1998 [17] | low | moderate | high | high | moderate | high | high | low | low |

**References**

[1] Garces P, Antoniades CA, Sobanska A, Kovacs N, Ying SH, Gupta AS, Perlman S, Szmulewicz DJ, Pane C, Nemeth AH, Jardim LB, Coarelli G, Dankova M, Traschutz A and Tarnutzer AA. Quantitative Oculomotor Assessment in Hereditary Ataxia: Discriminatory Power, Correlation with Severity Measures, and Recommended Parameters for Specific Genotypes. Cerebellum 2023. doi 10.1007/s12311-023-01514-8

[2] Alexandre MF, Rivaud-Pechoux S, Challe G, Durr A and Gaymard B. Functional consequences of oculomotor disorders in hereditary cerebellar ataxias. Cerebellum 2013: 12:396-405. doi 10.1007/s12311-012-0433-z

[3] Baloh RW, Konrad HR and Honrubia V. Vestibulo-ocular function in patients with cerebellar atrophy. Neurology 1975: 25:160-8. doi 10.1212/wnl.25.2.160

[4] Ciuffreda KJ, Kenyon RV and Stark L. Eye movements during reading: further case reports. Am J Optom Physiol Opt 1985: 62:844-52. doi 10.1097/00006324-198512000-00005

[5] Crane BT, Tian JR and Demer JL. Initial vestibulo-ocular reflex during transient angular and linear acceleration in human cerebellar dysfunction. Exp Brain Res 2000: 130:486-96. doi 10.1007/s002219900266

[6] Dale RT, Kirby AW and Jampel RS. Square wave jerks in Friedreich's ataxia. Am J Ophthalmol 1978: 85:400-6. doi 10.1016/s0002-9394(14)77738-4

[7] Ell J, Prasher D and Rudge P. Neuro-otological abnormalities in Friedreich's ataxia. J Neurol Neurosurg Psychiatry 1984: 47:26-32. doi 10.1136/jnnp.47.1.26

[8] Fahey MC, Cremer PD, Aw ST, Millist L, Todd MJ, White OB, Halmagyi M, Corben LA, Collins V, Churchyard AJ, Tan K, Kowal L and Delatycki MB. Vestibular, saccadic and fixation abnormalities in genetically confirmed Friedreich ataxia. Brain 2008: 131:1035-45. doi 10.1093/brain/awm323

[9] Fielding J, Corben L, Cremer P, Millist L, White O and Delatycki M. Disruption to higher order processes in Friedreich ataxia. Neuropsychologia 2010: 48:235-42. doi 10.1016/j.neuropsychologia.2009.09.009

[10] Furman JM, Perlman S and Baloh RW. Eye movements in Friedreich's ataxia. Arch Neurol 1983: 40:343-6. doi 10.1001/archneur.1983.04050060043006

[11] Hocking DR, Corben LA, Fielding J, Cremer PD, Millist L, White OB and Delatycki MB. Saccade reprogramming in Friedreich ataxia reveals impairments in the cognitive control of saccadic eye movement. Brain Cogn 2014: 87:161-7. doi 10.1016/j.bandc.2014.03.018

[12] Hocking DR, Fielding J, Corben LA, Cremer PD, Millist L, White OB and Delatycki MB. Ocular motor fixation deficits in Friedreich ataxia. Cerebellum 2010: 9:411-8. doi 10.1007/s12311-010-0178-5

[13] Luis L, Costa J, Munoz E, de Carvalho M, Carmona S, Schneider E, Gordon CR and Valls-Sole J. Vestibulo-ocular reflex dynamics with head-impulses discriminates spinocerebellar ataxias types 1, 2 and 3 and Friedreich ataxia. J Vestib Res 2016: 26:327-34. doi 10.3233/VES-160579

[14] Moschner C, Perlman S and Baloh RW. Comparison of oculomotor findings in the progressive ataxia syndromes. Brain 1994: 117 ( Pt 1):15-25. doi 10.1093/brain/117.1.15

[15] Ribai P, Pousset F, Tanguy ML, Rivaud-Pechoux S, Le Ber I, Gasparini F, Charles P, Beraud AS, Schmitt M, Koenig M, Mallet A, Brice A and Durr A. Neurological, cardiological, and oculomotor progression in 104 patients with Friedreich ataxia during long-term follow-up. Arch Neurol 2007: 64:558-64. doi 10.1001/archneur.64.4.558

[16] Spieker S, Schulz JB, Petersen D, Fetter M, Klockgether T and Dichgans J. Fixation instability and oculomotor abnormalities in Friedreich's ataxia. J Neurol 1995: 242:517-21. doi 10.1007/BF00867423

[17] Wessel K, Moschner C, Wandinger KP, Kompf D and Heide W. Oculomotor testing in the differential diagnosis of degenerative ataxic disorders. Arch Neurol 1998: 55:949-56. doi 10.1001/archneur.55.7.949
